# Effect of RNA quality on transcript intensity levels in microarray analysis of human post-mortem brain tissues

**DOI:** 10.1186/1471-2164-9-91

**Published:** 2008-02-25

**Authors:** Tatiana Popova, Detlev Mennerich, Andreas Weith, Karsten Quast

**Affiliations:** 1 Boehringer Ingelheim Pharma GmbH Co & KG, Birkendorfer Str. 65, Biberach and der Riss, Germany

## Abstract

**Background:**

Large-scale gene expression analysis of post-mortem brain tissue offers unique opportunities for investigating genetic mechanisms of psychiatric and neurodegenerative disorders. On the other hand microarray data analysis associated with these studies is a challenging task. In this publication we address the issue of low RNA quality data and corresponding data analysis strategies.

**Results:**

A detailed analysis of effects of post chip RNA quality on the measured abundance of transcripts is presented. Overall Affymetrix GeneChip data (HG-U133_AB and HG-U133_Plus_2.0) derived from ten different brain regions was investigated. Post chip RNA quality being assessed by 5'/3' ratio of housekeeping genes was found to introduce a well pronounced systematic noise into the measured transcript expression levels. According to this study RNA quality effects have: 1) a "random" component which is introduced by the technology and 2) a systematic component which depends on the features of the transcripts and probes. Random components mainly account for numerous negative correlations of low-abundant transcripts. These negative correlations are not reproducible and are mainly introduced by an increased relative level of noise. Three major contributors to the systematic noise component were identified: the first is the probe set distribution, the second is the length of mRNA species, and the third is the stability of mRNA species. Positive correlations reflect the 5'-end to 3'-end direction of mRNA degradation whereas negative correlations result from the compensatory increase in stable and 3'-end probed transcripts. Systematic components affect the expressed transcripts by introducing irrelevant gene correlations and can strongly influence the results of the main experiment. A linear model correcting the effect of RNA quality on measured intensities was introduced.

In addition the contribution of a number of pre-mortem and post-mortem attributes to the overall detected RNA quality effect was investigated. Brain pH, duration of agonal stage, post-mortem interval before sampling and donor's age of death within considered limits were found to have no significant contribution.

**Conclusion:**

Basic conclusions for data analysis in expression profiling study are as follows: 1) testing for RNA quality dependency should be included in the preprocessing of the data; 2) investigating inter-gene correlation without regard to RNA quality effects could be misleading; 3) data normalization procedures relying on housekeeping genes either do not influence the correlation structure (if 3'-end intensities are used) or increase it for negatively correlated transcripts (if 5'-end or median intensities are included in normalization procedure); 4) sample sets should be matched with regard to RNA quality; 5) RMA preprocessing is more sensitive to RNA quality effect, than MAS 5.0.

## Background

Analysis of microarray gene expression profiles of post-mortem brain tissues have become an important tool in studying neurodegenerative and psychiatric disorders [1-5]. In addition to a number of reported sets of differentially expressed genes specific to schizophrenia, Alzheimer's and Parkinson's diseases, autism, alcoholism etc. one can find continued discussions concerning quality and consistency of post-mortem brain analysis by microarray technology [6-12]. Main challenges in this area summarized in [13,14] are as follows: 1) limited availability of post-mortem material resulting in extreme diversity of subjects with respect to race, age, post-mortem interval, medication history, lifestyle and other variable factors; 2) complex character of brain tissues; 3) important expression changes in brain samples are often modest and concern low abundance genes; 4) the transcriptome is shaped by the treatment of the disease; 5) it is hard to separate the effect of disease from the normal progression of aging; 6) RNA integrity in post-mortem sampling can be influenced by pre-mortem and post-mortem events.

The limited availability of donor material may be one reason for difficulties in establishing proper experimental designs with respect to possible confounding factors. In order to address this issue a number of potential confounding factors and their effect on transcript abundances in the post-mortem brain samples have been intensively studied [15-22]. It is generally accepted that details of pre-mortem and post-mortem events effect the transcriptome [18,19], while real interrelations between expression level and confounders like donor age of death, pre-mortem hypoxia, agonal events and duration of agonal stage, brain pH, post-mortem interval before sampling, and RNA integrity are still under discussion [6,12,16,17,20]. Recently it has been shown that: 1) an increase of complexity and duration of agonal events causes an increase of variance of intensities and a decrease of between chip correlation [21], 2) differences in post-mortem interval before sampling leads to only marginal effects [16,17], and 3) differences in RNA quality leads to crucial effects that are much more pronounced than the disease (i.e. schizophrenia) related effects [22]. The latter point refers to the fact that the RNA quality of post-mortem brain samples depends on some difficult-to-control attributes. This results in a wide range of chip RNA quality in a sample set and finally in a strong signal reflecting RNA quality in the expression profiles. Thus, it is crucial to take these effects into account when analyzing microarray data derived from post-mortem brain tissues.

In this paper we present detailed analyses of the effects of RNA quality on measured transcript intensities and corresponding data analysis strategies in the context of post-mortem brain tissue microarray studies.

The main aim of our study was the analysis of genes deregulated in Parkinson's disease. On the basis of data derived from the GeneLogic database [23] gene expression profiles obtained with Affymetrix GeneChip platforms HG-U133_AB and HG-U133_Plus_2 were investigated. Sample sets for 10 different brain regions contained approximately 20–30 samples in each set (Table 1) and were hybridized to both chip platforms thus allowing high statistically confident conclusions to be drawn. RNA quality, which we have chosen to assess by 5'/3' ratios (see section 1 of Results and discussion) of the housekeeping genes beta actin (*ACTB*) and *GAPDH*, was found to introduce well-pronounced systematic noise into transcript expression levels. Some components of the noise have a clear biological basis. An algorithm to correct for the effect of RNA quality on the detected transcript intensities was investigated. As a measure of the quality of the correction procedure the correlation between the HG-U133_AB and HG-U133_Plus_2 derived expression profiles was used.

**Table 1:** Sample set sizes and data dependency structure

Chip platform		HG-U133_Plus_2.0		HG-U133_AB		Intersection	
		
		Controls	Parkinson's	Controls	Parkinson's	Controls	Parkinson's
**Brain tissue**	**Shortcut**						

Brodmann area 35	BA35	11	12	13	12	10	11
Caudate nucleus^**P**^	CauNuc	23	12	18	13	16	12
Nucleus ambiguus	NucAmb	12	12	13	12	11	11
Nucleus basalis of Meynert	NucBas	8	11	14	12	8	11
Pulvinar	Pulvinar	13	11	7	12	6	11
Putamen^**P**^	Putamen	17	11	13	13	10	11
Reticular formation of midbrain	RetForm	13	9	10	13	10	9
Septal area of paraterminal body of rhinencephalon	SepArea	25	12	12	12	10	12
Substantia nigra^**P**^	SubNig	11	13	10	12	7	12
Subthalamic nucleus^**P**^	SubthNuc	12	7	10	12	7	7

Summary of sample sizes per chip platform and brain tissue and data dependency structure with respect to donor subjects are presented. Intersection column shows number of samples sharing the same donor material. Brain tissue involved into Parkinson's disease marked by subscript ^**P**^.

In addition the contribution of a number of pre-mortem and post-mortem attributes to the overall detected RNA quality was investigated. In particular, the brain pH, duration of agonal stage, post-mortem interval before sampling and donor's age of death within considered limits were found to have no well pronounced and significant contribution.

## Results and discussion

### 1. RNA quality measures

In general the quality of mRNA material can be defined by the 28s/18s rRNA ratio, RIN or by some other less generally used indices [24,25]. Theoretically the RNA quality of brain samples should reflect the influences of clinical, pre-, and post-mortem events, if any. Basic *post chip *(after hybridization) RNA quality parameter commonly used in Affymetrix U133 chips is 3'/5' ratio of the housekeeping genes *GAPDH *(Glyceraldehyde-3-phosphate dehydrogenase) and *ACTB *(beta actin) which actually is RNA *degradation *index. This index reflects not only the original level of RNA integrity but also the accuracy of sample processing, namely RNA purification, reverse transcription, in-vitro amplification and labelling, fragmentation and hybridization. A low post chip RNA degradation index (3'/5' ratio < 3) corresponds to high quality material while high post chip RNA degradation index (3'/5' ratio > 3) could indicate both low quality material and/or problems encountered during sample processing. Correlation between 28s/18s rRNA ratio and 3'/5' ratio reported in recent publication [26] for post-mortem brain data is -0.64 for *GAPDH *and -0.48 for *ACTB*.

Historically 3'-end and 5'-end located probeset intensities of housekeeping genes were arranged into 3'/5' ratio and evaluated as a measure of transcript *degradation*. However, 3'/5' ratio is not good for computational purposes. First, it has singularity at zero in denominator which means irrelevant growth of values when 5'-end intensity goes down. Second, it is insensitive to changes in the low to moderate range of degradation especially if sample set contains both high and extremely low quality samples. And thirdly it is an RNA *degradation *measure while we would like to consider RNA *quality *measure (i.e. positively correlated to the quality itself). For these reasons we used the inverse ratio which is less popular but can also be used [6,10].

**Figure 1:**
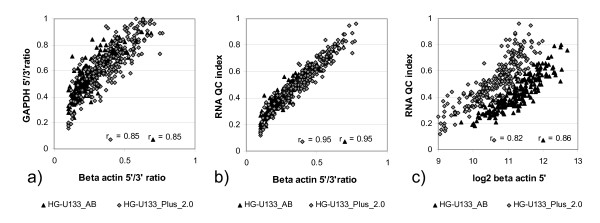
**Measures of RNA quality of Affymetrix GeneChip**. Measures of RNA quality of Affymetrix GeneChip by the example of Dataset 1: a) 5'/3' beta actin ratio vs 5'/3' *GAPDH *ratio; b) 5'/3' beta actin ratio vs RNA QC index; c) beta actin 5'-end abundance vs RNA QC index.

Thus, to characterise post chip RNA quality we use 5'/3' ratio of beta actin and *GAPDH *genes. The mean of two 5'/3' ratios is designated as *post chip *RNA quality (denoted further as RNA QC) of Affymetrix GeneChip sample in GeneLogic database. 5'/3' ratio of housekeeping genes and RNA quality vary from 0 to 1 with 0 stands for lower bound of RNA quality and 1 stands for upper bound of RNA quality. Figure 1 displays post chip RNA quality characteristics of considered post-mortem brain samples collected in Dataset 1. 5'/3' ratios and RNA quality are highly correlated with each other and also to 5'-end intensity of housekeeping genes. On the other hand median probeset intensity shows a rather poor correlation to RNA QC and 3'-end abundances have no correlation to RNA QC at all (data not shown). For further calculation the beta actin 5'/3' ratio was chosen and is further referred to as (post chip) RNA quality.

Overall, the RNA quality of the investigated data sets is rather poor and varies considerably. For this reason the effect of the RNA quality and methods of how to deal with it were investigated. Since Affymetrix microarrays tolerate RNA degradation due to the 3'-end biased probe set positions [16,27] it is reasonable to investigate even low RNA quality hybridizations given the value of the used material. A ratio of 0.3 for beta actin is recommended as the least acceptable value for post-mortem brain analysis with HG-U133_AB chips [6]. Nevertheless we decided to use a threshold of 0.1 to attempt to address the challenge of low quality chip data.

### 2. Correlation between transcript expression profiles and 5'/3' beta actin ratios

**Figure 2:**
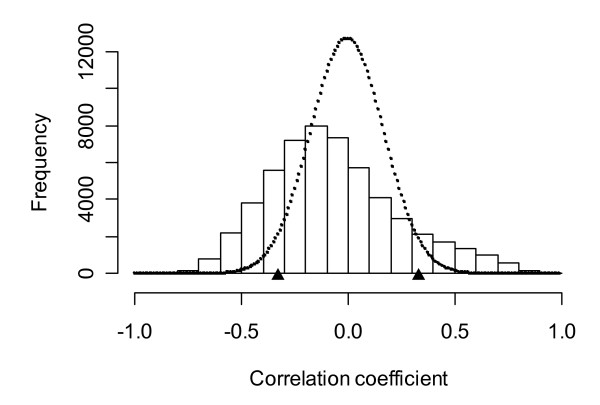
**Correlation structure caused by RNA quality**. Histogram of distribution of correlation coefficient between transcript expression profiles and beta actin ratio for septal area sample set HG-U133_Plus2.0 chip, normalization MAS 5.0. Dotted line indicates smoothed correlation coefficient distribution in randomised data. Triangles point to correlation coefficient of 0.05 significance level.

Correlation between the intensities of all transcripts and the corresponding beta actin ratios was investigated (Spearman rank correlation coefficient was used to avoid outlier effects). Figure 2 shows a typical distribution of correlation coefficients to beta actin ratio together with smoothed correlation coefficient density for randomised data. The bell-shaped density for randomized data indicates no correlation structure related to the beta actin ratio. As shown in Figure 2 two shapes differ dramatically. The real distribution displays significant asymmetry (mode = -0.2) and "heavy" tails.

**Figure 3:**
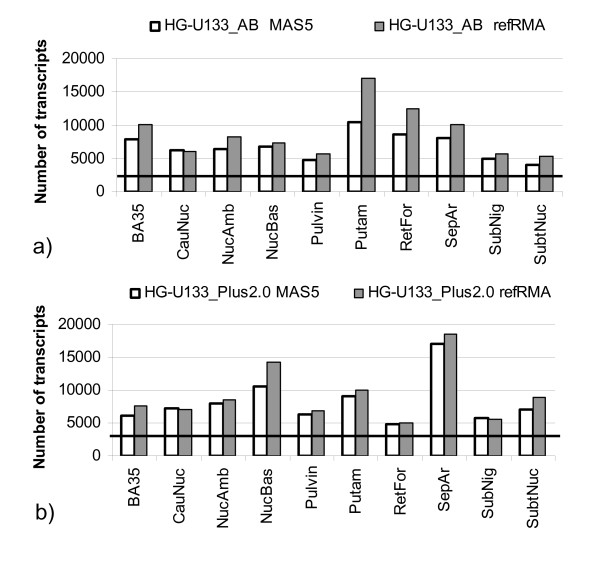
**Number of significant correlations to beta actin ratio within chip set**. Number of transcripts that show significant (<0.05) correlation to beta actin ratio in all considered sample sets for two chip platforms and two normalizations: a) HG-U133_plus2.0 chip, b) HG-U133_AB chip. Horizontal line indicates a number of significant hits expected by chance. They are: 5% of 54613 ≅ 2731 for HG-U133_Plus2.0 chip and 5% of 44792 ≅ 2240 for HG-U133_AB chip.

Figure 3 shows the number of transcripts exhibiting expression profiles significantly (p ≤ 0.05) correlated to the beta actin ratio in all sample sets under consideration. In all considered cases the number of RNA quality dependent transcripts is higher than expected by chance (horizontal line in Figure 3 corresponds to 5% of all transcripts represented on the chip). The fact that up to 30% of all transcripts display expression profiles correlated to the beta actin ratio implies that RNA quality can act as a major source for the previously reported correlation structure in microarray data [28]. refRMA normalized data seems to be more sensitive to the RNA quality effect compared to MAS 5.0 data (Figure 3).

Two observations are striking: 1) number of RNA quality dependent transcripts differ significantly within tissue sets and chip platforms; 2) all distributions of correlation coefficient (similar to one shown in Figure 2) display significant asymmetry: transcripts which correlate negatively to RNA quality are predominant.

**Figure 4:**
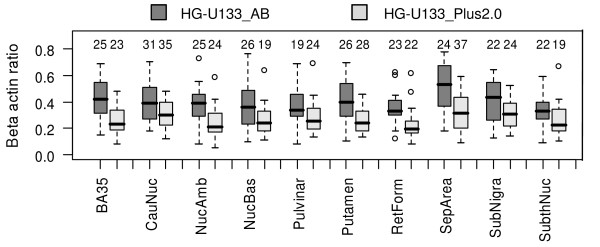
**RNA quality in considered sample sets**. Box plots characterizing the range of beta actin ratio in tissue sample sets accompanied by size of the sample set: chip HG-U133_AB data are in dark grey, chip HG-U133_Plus_2.0 data are in light grey; sample set sizes are indicated above the box plots.

Differences in the distributions and hence the number of RNA-quality dependent transcripts may originate from a number of sources. Among others it could be the sample size or the range of beta actin ratios in the investigated sample sets (Figure 4). However, there was no clear dependency found among sample size, beta actin ratio distribution, and the number of RNA-quality dependent transcripts within a sample set.

**Figure 5:**
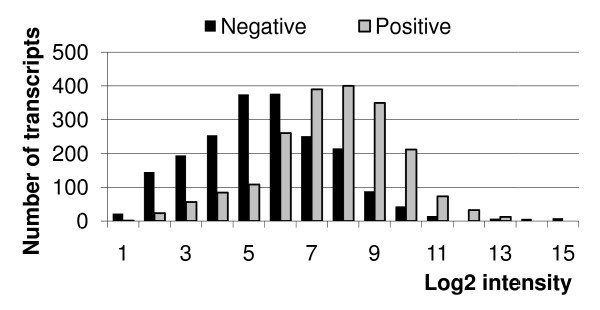
**Positive and negative correlation to RNA quality and transcript abundances**. Distribution of abundances of the top 2000 positively and top 2000 negatively correlated to beta actin ratio transcripts out of 7035 significant (*p *≤ 0.05) ones for subthalamic nucleus sample set chip HG-U133_Plus_2. Other sample sets show similar behavior.

Another interesting feature of the distributions of correlation coefficients identified in this study is the significant predominance and strength of negative correlations between transcript intensity and post chip RNA quality. In order to investigate the reason for this finding the overall intensity of transcripts with pronounced positive or negative correlation was examined. As an example, a barchart containing the top 2000 negatively (-0.97 < r < -0.66) and top 2000 positively (0.6 < r < 0.97) correlating transcripts out of 7035 significant (*p *≤ 0.05) ones for subthalamic nucleus sample set is shown in Figure 5. A clear separation of the two distributions occurs. Negative correlations are shifted to low intensity transcripts whereas positive correlations are mainly exhibited by high abundance transcripts.

Transcripts which show a low-intensity signal (<5–6 in log2 scale) are either not expressed or low expressed ones. Negative correlation to RNA quality means that the worse the quality the stronger the signal becomes. One possible reason for this effect in the case of unexpressed transcripts is the enrichment of low RNA quality material with fragments of degraded mRNA. Fragments of degraded RNA could subsequently increase low intensity signals of unexpressed transcripts on low quality chips by nonspecific hybridization. Another possible reason is related to the commonly used normalization strategies. In the case of a decrease in the abundance of actual expressed transcripts due to the decrease of RNA quality the relative level of low signals increases. This also holds true for MAS 5.0 preprocessing due to the scaling step as RMA (or refRMA) due to the quantile normalization.

**Figure 6:**
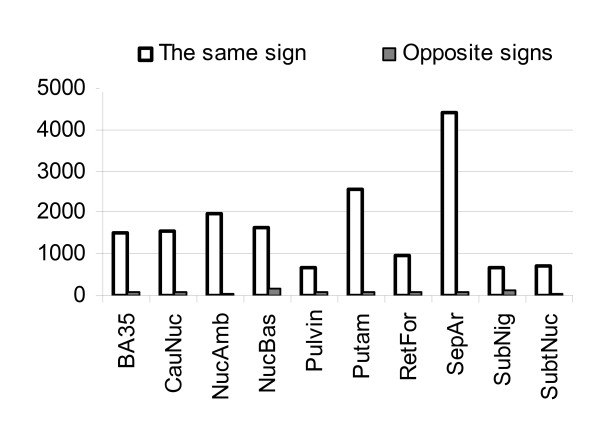
**Inter-platform consistency of RNA quality dependency**. Number of consistently (the same positive or negative sign) and inconsistently (opposite sign) correlated to RNA quality transcripts among ones found to be significant in two hybridizations. Comparison with Figure 3 shows that in general, between 5% and 20% of RNA quality dependent transcripts have significant correlation (p < 0.05) of the same sign on both chips. Hence the majority of correlations appeared to be dependent on current hybridization.

Taking advantage of the availability of the two chip platforms and numerous sample sets we could check the stability of the observed correlations i.e. their consistency and reproducibility in different experiments. The number of consistently (the same positive or negative sign) and inconsistently (opposite sign) correlated to RNA quality transcripts among ones found to be significant in two hybridizations are shown on Figure 6. In general between 5% and 20% of RNA quality dependent transcripts showed significant correlation (p < 0.05) of the same sign for both chips. Hence the majority of correlations are dependent on the current hybridization.

Basic conclusions to be made from this data are 1) a correlation structure in microarray data exist which is determined by post chip RNA quality; 2) within a sample set significantly different levels of dependency to RNA quality may occur; 3) the majority of transcripts show negative correlation to RNA quality. Since from 10% to 30% of all transcripts present on the chip may correlate to RNA quality the second point is explicitly stressed here. RNA quality can strongly affect the results of the experiment as described previously [22] but this is not necessarily the case. As an example from our current study, *Septal area *(not involved into Parkinson's disease) seemed to show strong deregulation related to Parkinson's disease which appeared to result from high levels of noise introduced by RNA quality. In contrast *Caudate nucleus *(involved into the disease) showed moderate dependency on RNA quality and strong deregulation related to Parkinson's disease.

It is important to note that the third point concerns data normalization procedures relying on housekeeping genes. The 3'-end intensities of housekeeping genes show poor correlation to the RNA quality in considered datasets. Hence, this normalization does not influence the overall correlation structure introduced by RNA quality. It might be a reason why normalization on housekeeping genes did not eliminate the correlation structure mentioned previously in [28] as an open point. Including median or 5'-end intensities into normalization procedure will increase the systematic noise for those transcripts exhibiting negative correlation to RNA quality.

### 3. Principal components, explained variance and the source of RNA quality dependencies

**Figure 7:**
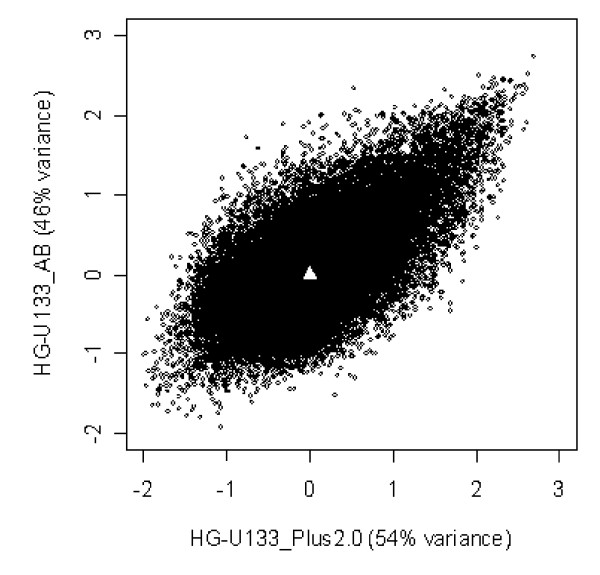
**Principal component analysis of brain tissue correlation profiles**. Principal component analysis of 10-dimentional vectors of correlation coefficients between transcript expression profile and beta actin ratio was performed. The first principal component scores of 44792 common transcripts (HG-U133_Plus_2.0 chip versus HG-U133_AB chip) are shown. Positive scores correspond to positive correlation to beta actin ratio and negative scores correspond to negative one. Points with top positive and top negative scores demonstrate consistent RNA quality dependency through 10 brain tissues and 2 chip hybridizations.

Since the majority of expressed transcripts are brain specific it is reasonable to investigate between tissue consistency of the RNA quality effect. Consider all 10 brain sample sets and their correlation profiles to RNA quality. Let each transcript be characterized by a 10-dimensional vector of correlation coefficients between its intensity and the 5'/3' beta actin ratio. Summary of principal component analysis of these vectors is presented in Table 2. The first principal component is prevailing for both chip platforms. Moreover, transcripts with high first principal component score are correlated to RNA quality with the same sign in all considered brain tissues. Figure 7 shows consistency of the first principal component scores of two chip platform data (the sign of the scores correspond to the sign of correlation to RNA quality).

**Table 2:** Summary of principal component analysis

	HG-U133_AB chip				HG-U133_Plus2.0 chip			
	
	PC_1	PC_2	PC_3	PC_4	PC_1	PC_2	PC_3	PC_4
Proportion of variance, %	46	12	8	6	54	7	7	5
Eigenvalue	4.55	1.24	0.80	0.62	5.37	0.73	0.69	0.60

Summary of principal component analysis of 10-dimensional vectors of correlation coefficients between transcript intensity and the 5'/3' beta actin ratio. The first four principal components out of 10 possible are shown. The first principal component is prevailing for both chip platform data.

**Figure 8:**
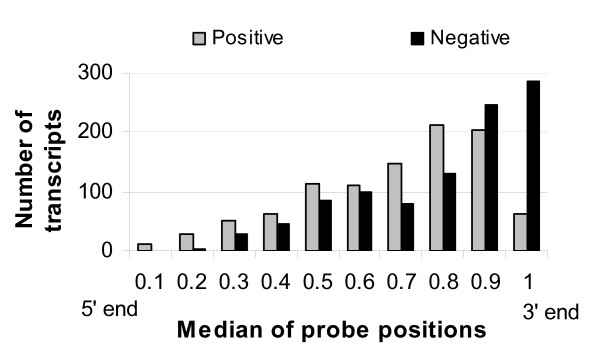
**Positive and negative correlation to RNA quality and median of relative probe positions**. Distribution of the median of relative probe positions for 1000 top positive score transcripts and 1000 top negative score ones. The first principal component scores were summarised for two chips. Positively correlated to RNA quality transcripts are underrepresented in the very 3' end of probes position distribution, whereas negatively correlated transcripts are overrepresented in the 0.8–1 interval.

**Figure 9:**
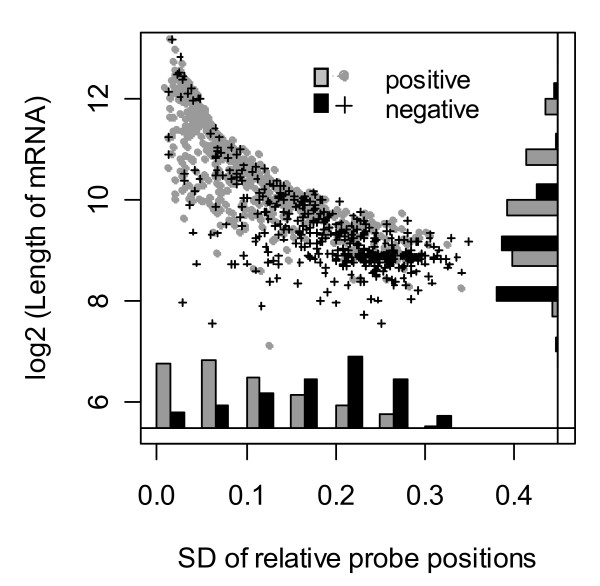
**Positive and negative correlation to RNA quality and the length of mRNA**. Scatter plot and marginal distributions of the standard deviation of relative probe positions versus mRNA length for positively and negatively correlated transcripts exhibiting a median of relative probe positions in the range of 0.1–0.8. 400 positively and 600 negatively correlated transcripts out of 2000 presented in Figure 8 are shown.

Consistent dependencies of transcripts on RNA quality among a number of experiments imply the presence of transcript specific components of the effect. More detailed investigations revealed that the sign of correlation to RNA quality is partially determined by distribution of the probes in the mRNA (median of relative probe positions and their standard deviation), the length of the mRNA, and probably overall stability of mRNA species (Figure 8, 9). Figure 8 displays the distribution of medians of relative probe positions in the mRNA for 1000 top positive score transcripts and 1000 top negative score ones. Negatively correlated transcripts are overrepresented in the very 3'-end of relative probe positions, whereas positively correlated transcripts have a shift in relative probe positions towards the 5'-end direction. This finding corresponds to the fact that RNA degradation predominantly starts at the 5'-end. The better the RNA quality, the higher the measured signal of the 5'-end located probe set. Correspondingly, the worse the RNA quality, the lower the level of 5'-end transcripts, the higher the relative level of 3'-end transcripts, since the overall amount of RNA in the experiment is always the same.

The mixture of positively and negatively correlated transcripts in the range of 0.1–0.8 of the median of relative probe positions (see Figure 8) was considered separately. Figure 9 shows a scatter plot of the standard deviation of relative probe positions versus the length of the corresponding mRNA together with their marginal distributions. A difference in marginal distributions between positively and negatively correlated transcripts is clear. The observed difference in standard deviation again reflects the conception of 5'-end to 3'-end RNA degradation. High standard deviations of relative probe positions such as displayed in Figure 9 usually correspond to probe sets containing at least some probes close to the 3'-end. The presence of probe(s) at the 3'-end can be a reason for observing negative correlation to RNA quality.

Stability of mRNA species can also play an important role in this context. Profiles of relatively unstable transcripts should be positively correlated to RNA quality since their overall signal intensity is higher for chips with good RNA quality. Stable transcripts should exhibit negative correlation to RNA quality because they are enriched in poor RNA quality samples.

However there exist no mRNA stability index and the whole conclusion cannot be verified. There is some evidence that short mRNA species are more stable [29]. As displayed in Figure 9 short transcripts are overrepresented among transcripts exhibiting negative correlation to RNA quality.

Using predicted mRNA stability data from a T lymphocyte study [30] (obtained with HG-U95A GeneChip) and the set of predicted list of mRNAs with AU-rich elements in 3'- or 5'-end UTRs [31] which are supposed to be unstable [12,17] we were not successful in obtaining a clear picture supporting the conclusion. The reason for this difficulty is mainly related to the high level of noise and irrelevance in considered mRNA lists. Although some trends could be observed their significance could hardly be proved.

In summary, the consistent transcript-dependent components of expression profile dependency on post chip RNA quality can be explained by the distribution of relative probe positions, the length of the mRNA, and probably the stability of the mRNA species.

It should be noted that the beta actin ratio as a measure of RNA quality definitely focuses on transcripts subjected to a certain type of degradation. Apparently other measures can be determined which show other sets of "RNA quality dependent" transcripts. However, using the beta actin ratio measure, we revealed a strong correlation structure within the Affymetrix GeneChip gene expression data. As a consequence more reliable data analysis strategies could be applied.

### 4. Data correction procedure based on a linear model

In general confounding factors are dealt with by including them in a linear model. In cases where the confounder correlates to a feature of interest, removing the confounder would result in elimination of, or at least reduction in, the signal obtained. Some of the sample sets under consideration in the current study are unbalanced with respect to RNA quality. Therefore it becomes impossible to separate the effect of RNA quality from the effect of the disease. In order to avoid reducing the Parkinson's signal a linear model on the basis of the control group was introduced. Finally this model was utilized to correct the RNA quality effect of both Parkinson's and control data (see Methods).

Since expression profiles are available for two chip platforms and probe sets detected by these platforms largely overlap we used the inter-platform correlation as a quality measure for the applied correction procedures. Expression profiles generated by using material stemming from the same set of donors should correlate if they are related to actually present transcripts and should show no correlation in the case of actually absent transcripts. On the other hand systematic noise is introduced by the RNA quality. Between 5000 and 16000 transcripts were found to be RNA quality dependent on HG-U133_Plus_2.0 chip and up to 20% of them showed the same behavior on HG-U133_AB chip while others did not. Thus RNA quality could inflate irrelevant correlations and suppress relevant ones.

Post chip RNA quality profiles themselves should display inter-chip set correlation if the input of original sample quality is constitutive and sample processing introduces modest random fluctuations. Therefore an appropriate correction procedure should reconstitute the perturbed real inter-chip set correlations of expression profiles and eliminate the irrelevant ones.

A summary of correcting procedures applied to all 10 considered sample sets is presented in Table 3. Correlations of post chip RNA quality profiles are also shown. Small sample size and low overall RNA quality have led to an absence of inter-platform correlation of RNA quality profiles for some sample sets. However, the obtained results are comprehensible (see Table 3): 1) for sample sets having not correlated RNA quality profiles the inter-platform correlations increase on average; 2) for samples containing coherent profiles inter-platform correlations decrease on average. This can be taken as evidence for a real elimination of noise by the procedure. However, it only holds true for the set of transcripts that are consistently dependent on RNA quality. Application of the correction procedure to two chip platforms independently shows an average decrease of between platform correlations for all sample sets. Low generalization ability of the linear model based on control groups appears most probably due to the small sample sizes. Thus, one should cautiously apply the data correction procedure in analogous settings. Nevertheless, taking into consideration RNA quality dependency of transcripts can help in reducing the number of false positive hits in analysing gene deregulation.

**Table 3:** Outcome of linear correction procedure for RNA quality

Brain tissues	Correcting consistent transcripts only		Correcting HG-U133_AB and HG-U133_Plus2.0 data independently		Inter-chip coherence of RNA quality profiles
		
	Increased	Decreased	Increased	Decreased	
Brodmann area 35	684	715	1355	2467	0,67
Caudate nucleus	544	688	1062	2164	0,48
Nucleus ambiguus	849	1367	754	2924	0,79
Nucleus basalis *	1013	526	1690	3089	0,01 *
Pulvinar *	675	442	1301	2401	-0,11 *
Putamen	962	1331	729	3390	0,74
Reticular formation	385	389	865	2329	0,37
Septal area	1185	2698	1175	6587	0,80
Substantia nigra *	685	257	808	1935	-0,13 *
Subthalamic nucleus	588	704	1454	3771	0,43

Number of transcripts with corrected expression profiles exhibiting increase and decrease in inter-platform correlation after linear correction for RNA quality. Linear correction was performed in two variants: 1) correcting profiles which show significant (p < 0.05) RNA quality dependency of the same sign on both chips (consistent transcripts), and 2) correcting all significantly correlated (p < 0.05) to RNA quality profiles in two chips independently. The last column shows coherency of RNA quality profiles for two chip datasets. Sample sets with not correlated RNA quality profiles are marked by asterisk.

One of the major tasks when analyzing brain tissue using microarrays is detecting changes in low-abundant genes [6]. Inter-chip set correlation can be utilized as a criterion to distinguish low signals from the noise. In order to eliminate false correlations and reveal true ones correction for RNA-quality effects becomes extremely important.

### 5. Details of pre-mortem and post-mortem events and RNA quality

Donor age of death, pre-mortem hypoxia, other agonal events, duration of agonal stage, brain pH, post-mortem interval before sampling, and RNA integrity were previously described as important factors related to data consistency in the context of microarray analysis of post-mortem brain samples [18,19,21]. Biological and computational experiments showed some particular and general effects of these parameters on RNA integrity and microarray gene expression data. However, a deeper understanding of the details is still missing. Since a strong effect of RNA quality was revealed in our study the next task consisted of investigating the contribution of pre-mortem and post-mortem details to the post chip RNA quality.

In these analyses dataset 2 was introduced which contained donors with known age of death, duration of agonal stage, brain pH, and post-mortem interval before sampling. Since pre-mortem hypoxia was difficult to determine it was not considered here. In any case, all samples were taken post-mortem and thus tissues are assumed to have suffered from hypoxia. Due to the lack of variety in agonal events in the given sample set this feature was also not considered.

**Figure 10:**
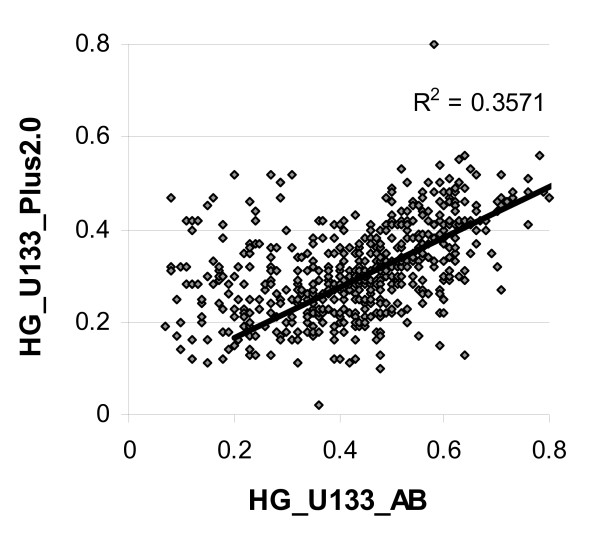
**Consistency of chip RNA quality for two chip hybridizations**. Scatter plot of the beta actin ratios of paired samples hybridized to two Affymetrix chip platforms (a mixture of brain tissues). Line shows the linear trend (*r *= 0.6) calculated on the basis of data points in the interval 0.3 – 1 of HG-U133_AB beta actin ratio.

A scatter plot of paired beta actin ratios for HG-U133_AB and HG-U133_Plus_2 data sets is shown in Figure 10. An obvious linear trend in the 0.3 – 1 interval of HG-U133_AB beta actin ratio (*r *= 0.53) can be seen. This is an indication of the existence of a systematic component of RNA quality that is independent of the actual chip type.

**Figure 11:**
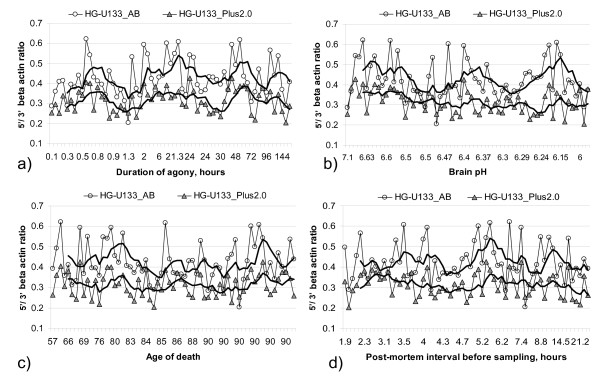
**Chip RNA quality versus some pre-mortem and post-mortem attributes**. Median of beta actin ratios in a sample set related to the same donor versus donor pre-mortem and post-mortem attributes. Each point represents a single donor. Each sample set contains samples derived from various brain areas of one donor. Donors (x-axis labels) are ordered by corresponding parameter, 5 point moving average trend lines are shown. A barely perceptible decrease in trend line is present with decreasing brain pH (*r *= 0.26), but only for HG-U133_Plus_2.0 chip data.

Dataset 2 contains from 3 to 19 brain tissue samples related to each of 63 donors with known pre- and post-mortem history. The medians of beta actin ratios related to the same donor were calculated and considered as a function of known pre-mortem and post-mortem details. Results are shown in Figure 11. No evidences for the dependence of chip RNA quality on brain pH, donor age of death, duration of agony stage, and post-mortem interval before sampling was found. There is no pronounced bias in the trend lines displayed in Figure 11. Also no joint effect of mentioned parameters was found. Hence, it can be concluded that the investigated details of pre-mortem and post-mortem history do not contribute primarily and significantly to the post chip RNA quality in the described setup (see Conclusions).

Brain pH is mentioned as a factor of importance for RNA quality in some publications [20]. Even though a slow decrease of the beta actin ratio trend line with decreasing brain pH can be detected in the case of the HG-U133_Plus_2 chip dataset (Figure 11b; *r *= 0.26) this cannot be accept as a strong evidence. As displayed in the same figure the HG-U133_AB chip data does not follow the same trend.

Some bias of trend lines for post-mortem intervals more than 9 hours can also be observed. In general one should expect expression levels of at least some transcripts to depend on post-mortem interval before sampling. For example, some mRNA species in brain tissues were reported to have short half lives, degrading within the first 2–3 hours after death [17,18]. However, beta actin ratio does not reflect these changes.

Direct influences of post-mortem intervals on the signal values can be examined by the same strategy as in section 3. One would expect predisposed mRNA species to be affected in the same way during the time before sampling through all brain tissues. However, the first principal components of the correlation coefficients between signal values and post-mortem intervals exhibited only marginal inter-tissue consistencies (data not shown). An important fact in this regard maybe a bias in the sample sets. Only very few samples in Dataset 1 show a post-mortem interval of 2–3 hours or less while others were sampled more than 5 hours after death (m ± *σ *is 8 ± 6 hours). Probably all mRNA species with a short half-life period are already degraded at the moment of sampling whereas stable mRNAs stay constant within this time interval. This may be the reason why no systematic component in the dependency of transcript intensity on post-mortem interval before sampling was found.

## Conclusion

We performed a detailed analysis of effects of post chip RNA quality on the measured abundance of transcripts in post-mortem brain samples. The contribution of a number of pre-mortem and post-mortem attributes to the overall detected RNA quality effect was investigated. Such parameters as brain pH, duration of agonal stage, post-mortem interval before sampling and donor's age of death within considered limits were found to have no well-pronounced and significant contributions to post chip RNA quality.

In a recent publication [26] a number of quality parameters related to microarray studies of post-mortem brains were considered on the basis of 90 brain samples. This data supports our conclusions and gives some additional insights into the problem. For example, sample RNA quality measured by 28s/18s rRNA ratio is correlated (1) to post chip RNA quality measured by 3'/5' ratio of *GAPDH *and *ACTB *and (2) to integral Affymetrix chip characteristics as percent of present calls and scaling factor (MAS 5.0). On the other hand pre- and post-mortem parameters, namely, agony factor score, brain pH and post-mortem interval, do correlate to percent of present calls and scaling factor but *do not correlate *to RNA quality measures.

These findings suggest that the input of pre- and post-mortem parameters in the post chip RNA quality signal is rather weak. On the other hand post chip RNA quality input in expression profile is very strong because the 3'/5' ratio is directly related to measured intensities. Therefore our data suggests that in microarray analysis of post-mortem brains one should 1) collect all pre- and post-mortem parameters together with microarray sample; 2) check pre- and post-mortem parameters influence directly by considering expression profiles; 3) use this information in analysis of relevance of found deregulated gene set.

In current study we revealed the detailed profile of post chip RNA quality effect and investigated its technological and biological origin. RNA quality was found to introduce well pronounced systematic noise into signals obtained from microarray analysis of post-mortem brains. Between 10% and 30% of all transcripts in a chip correlate to RNA quality. According to this study RNA quality effects have: 1) a "random" component which is introduced by the technology and 2) a systematic component which depends on the features of the transcripts and probes. Random components mainly account for numerous negative correlations of low-abundant transcripts. These negative correlations emerge from non-specific hybridization and the biased data-normalization technology. They are not reproducible and are mainly introduced by an increased relative level of noise. RMA (or refRMA) preprocessing seems to be more sensitive to the effect than MAS 5.0 preprocessing. This finding can be explained by the quantile normalization step.

Three major contributors to the systematic noise component were identified: the first is the probe set distribution, the second is the length of mRNA species, and the third is the stability of mRNA species. Positive correlations reflect the 5'-end to 3'-end direction of mRNA degradation whereas negative correlations result from the compensatory increase in stable and 3'-end probed transcripts. Systematic components affect the expressed transcripts by introducing irrelevant gene correlations and can strongly influence the results of the main experiment.

A linear model correcting the effect of RNA quality on measured intensities was investigated. If the set of consistently dependent on RNA quality transcripts is corrected, subtraction of linear RNA quality effects results in an average increase of inter-platform correlation. This is the evidence of real elimination of noise by the procedure. Application of the correcting procedure to two chip platforms independently shows an average decrease of between platform correlations. So, one should cautiously apply the data correction procedure. Nevertheless, taking into consideration RNA quality dependency of transcripts can help in reducing the number of false positive hits in analysing gene deregulation.

Using the 5'/3' beta actin ratio, we revealed a strong correlation structure within the Affymetrix GeneChip gene expression data. As a consequence more reliable data analysis strategies could be applied. The following points are worth mentioning in cases with a wide range of post chip RNA quality in considered sample set: 1) testing for RNA quality dependency should be included in the preprocessing of the data; 2) investigating inter-gene correlation without regard to RNA quality effects could be misleading; 3) data normalization procedures relying on housekeeping genes either do not influence the correlation structure (if 3'-end intensities are used) or increase it for negatively correlated transcripts (if 5'-end or median intensities are included in normalization procedure); 4) sample sets should be balanced with regard to RNA quality.

## Methods

### Datasets

GeneLogic has collected gene expression profiles stemming from various different tissues and cells accompanied by an exhaustive number of clinical parameters. These data cover key therapeutic areas such as oncology, inflammation, cardiovascular disease, disorders of the central nervous system and metabolic disorders [23].

### Dataset 1

Since Parkinson's disease was the main subject of the current study sample sets representing ten different brain regions were included. Four of them are primarily involved in Parkinson's disease whereas six regions are located spatially close to the former ones and act as a control to detect non specific deregulation (see Table 1).

The main criteria for donor selection were: 1) presence of Parkinson's disease and absence of other neurodegenerative disorders for experimental groups; 2) absence of neurodegenerative and psychiatric disorders and an age of death greater than 50 years for the control groups.

The main criteria for sample selection concerning data quality were: 1) minimum value of between chip correlation within a single tissue sample set should be greater than 0.8; 2) mean beta actin and *GAPDH *5'/3' ratio should be greater than 0.1 (see details in Results and discussion, section 1). Due to low between chip correlation less than 2 outlying chips were filtered out from each group. Filtered chips are real outliers as they have low correlation with *all *other chips. Due to the second criterion 3–4 chips with extremely low RNA quality were excluded from the whole dataset.

Expression profiles obtained with two different Affymetrix GeneChips platforms, HG-U133_AB and HG-U133_Plus_2.0, were available for analysis. Comparison of the measured expression intensities of the two chip platforms was performed on the basis of 44792 common probesets. Due to intersecting sets of donors (see Table 1 for an overview) the sample sets are partially dependent. This dependence mainly concerns the Parkinson's groups which contain samples obtained from 18 different subjects only. Control groups are more independent consisting of material stemming from 65 different donors.

GeneLogic's database contains pre-calculated MAS 5.0 and refRMA [32] normalized expression values. refRMA is a generalization of the RMA normalization method that utilizes the set of reference samples as an independent basis for quantile normalization. MAS 5.0 normalized data were used by default and refRMA data were used for comparison. Beta actin and *GAPDH *5'/3' ratios were calculated from initial data, all further calculations were performed with log2 transformed intensities.

In this paper we describe the effects of RNA quality on the measured RNA abundances and whether the RNA quality itself resulted from pre-mortem and post-mortem events in the donor's history. The effects of donor gender and donor age in microarray analysis of human brains were found to be much less pronounced than the effects of RNA quality [16] and are not considered here in detail. Material used in our analyses was taken from approximately equal numbers of male and female donors. We focussed on subjects with an age of death in the range of 51 – 90 years.

Each expression profile was characterised by the following set of parameters:

1. binary variable for disease that equals 1 for donors suffering from Parkinson's disease and 0 for donors not suffering from Parkinson's disease,

2. beta actin levels (AFFX-HSAC07/X00351_5_at, _M_at and _3_at probe set intensities) and beta actin ratio, calculated as ratio of _5_at and _3_at probe sets intensities,

3. *GAPDH *levels (AFFX-HUMGAPDH/M33197_5_at, _M_at and _3_at probe set intensities) and *GAPDH *ratio calculated as ratio of _5_at and _3_at probe sets intensities,

4. RNA quality index (RNA QC) that is mean 5'/3' ratio of beta actin and *GAPDH*,

5. post-mortem interval before sampling,

6. brain pH,

7. duration of agony stage.

Since information about brain pH and duration of agony stage is not available for the majority of donors in Dataset 1, an additional dataset (Dataset 2) was compiled.

#### Dataset 2

Dataset 2 consists of all brain tissue samples with known donor brain pH and duration of agony stage contained in the database. These samples belong to various brain regions (19 in total). Donor's age of death was restricted to be in the same range as in Dataset 1 (from 51 to 90 years). Overall, Dataset 2 includes data derived from 63 donors with 3–19 Affymetrix GeneChip expression profiles each.

### Linear model

The observed normalised (MAS5 or RMA) log_2 _intensity *Y*_*ijk *_of *i*th sample and *j*th probe-set corresponding to chip platform *k *is modelled by the following linear equation:

*Y*_*ijk *_= *α*_*jk *_+ *β*_*jk*_*R*_*ijk *_+ *ε*_*ijk*_, *i *= 1,..., *M*_*k*_, *j *= 1,..., *N*_*k*_, *k *= 1, 2

where *α*_*jk *_represents an interception, *β*_*jk *_is a slope, *R*_*ijk *_stands for corresponding RNA quality measure namely, 5'/3' beta actin ratio, *ε*_*ijk *_is an error assumed to be normally distributed with zero mean, *N*_*k *_is a number of transcripts on GeneChip (*N*_1 _= 44792 corresponds to HG-U133_AB platform and *N*_2 _= 54613 corresponds to HG-U133_Plus_2.0), and *M*_*k *_is number of chip samples. The model is fitted independently for two chip platforms based on observations related to Control group.

Having estimated interception ${\hat{\alpha }}_{jk}$ and slope ${\hat{\beta }}_{jk}$ on the basis of control group, the whole dataset is corrected as follows:

$$Y_{ijk}^{C}=Y_{ijk}-{\hat{Y}}_{ijk}=Y_{ijk}-({\hat{\alpha }}_{jk}+{\hat{\beta }}_{jk}R_{ijk}).$$

Here subtraction of RNA quality effect is equivalent to consideration of model errors instead of initial values.

5'/3' beta actin ratio close to 1 correspond to ideal quality chip data. Using linear model one can calculate estimation of baseline transcript intensity obtained on ideal quality chip:

$$Y_{jk}^{I}={\hat{\alpha }}_{jk}+{\hat{\beta }}_{jk}\cdot 1.$$

Thus corrected data of baseline intensity level *Y*^*I*^_*jk *_can be obtained:

$$Y_{ijk}^{C}=Y_{jk}^{I}+(Y_{ijk}-{\hat{Y}}_{ijk})=Y_{ijk}+{\hat{\beta }}_{jk}(1-R_{ijk}).$$

Linear model correction is performed for those transcripts that are considered to be RNA quality dependent (with significant correlation to RNA quality) either for one chip platform or for both.

### Assessment of quality of correcting procedure

Correlation between corrected expression profiles {*Y*^*C*^_*ij*1_}_*i *= 1,..., *M *_and {*Y*^*C*^_*ij*2_}_*i *= 1,..., *M *_(where *M *is the number of paired samples) was compared to correlation between initial expression profiles {*Y*_*ij*1_}_*i *= 1,..., *M *_and {*Y*_*ij*2_}_*i *= 1,..., *M *_on the basis of sample sets completed by the same donors and for those transcripts *j *which are common for two chip platforms. The shift in correlation was taken into account if either correlation after correction or correlation before correction were not less than 0.3 (in order to ignore not expressed and noisy profiles). Correcting HG-U133_AB and HG-U133_Plus2.0 data independently means that each profile correlated to RNA quality with <0.05 significance level was corrected. Correcting consistent transcripts means correcting only those profiles that show RNA quality dependency of the same sign with significance <0.1 on both chips.

## Authors' contributions

TP carried out the main computational study and drafted manuscript. DM participated in analysis of computational results. AW supervised the project. KQ contributed to design and coordination and helped to draft the manuscript. All authors read and approved the final manuscript.
